# 

**Published:** 1899-04

**Authors:** 


### SELECTIONS.

Article I.—A Last Word. E. K. Wedelstaedt........ 529
" II.—Hemorrhage After Fxtraction........ 535
" III.—Continuous-Gum Work........ 537
" IV.—Report of a Case of Apparent Disease of Antrum of Highmore Dr. W. H. Morgan........ 542
" V.—Education of the Public. A. T White, D. D. S........ 546
" VI.—Dental Education of the Public........ 548
" VII.—The Medicinal Prevention of Dental Decay. George Howe Winkler, M. D, D. D. S........ 550
" VIII.—Infection of the Facial and Cervical Lymphatic Ganglia as a Result of Dental Lesions. Chas. B. Porter, D. D. S........ 557
" IX—Ethyl Bromide as an Anæsthetic. E. B. Dickinson, D. D. S........ 560

### COMMUNICATED.

Seventh District Dental Society of New York........ 568

### EDITORIAL, ETC.

Dental Department University of Maryland Commencement........ 569
Baltimore College of Dental Surgery Commencement........ 571

### MONTHLY SUMMARY.

Death From Cutting a Wisdom Tooth........ 573

Removal of Green Stain........ 573

A Tube-Dowel Crown........ 574

Temporary Sets of Teeth........ 575

Karolit........ 576

